# Correlation Consistent Basis Sets and Core Polarization Potentials for Al–Ar with ccECP Pseudopotentials

**DOI:** 10.1021/acs.jpca.2c04446

**Published:** 2022-08-17

**Authors:** Adam N. Hill, Anthony J. H. M. Meijer, J. Grant Hill

**Affiliations:** Department of Chemistry, University of Sheffield, Sheffield S3 7HF, United Kingdom

## Abstract

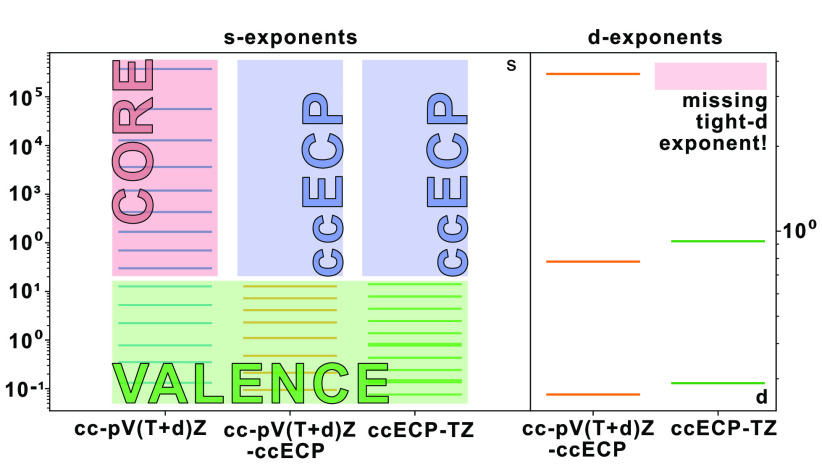

New correlation consistent basis sets for the second-row atoms (Al–Ar) to be used with the neon-core correlation consistent effective core potentials (ccECPs) have been developed. The basis sets, denoted cc-pV(*n*+d)Z-ccECP (*n* = D, T, Q), include the “tight”-d functions that are known to be important for second-row elements. Sets augmented with additional diffuse functions are also reported. Effective core polarization potentials (CPPs) to account for the effect of core–valence correlation have been adjusted for the same elements, and two different forms of the CPP cutoff function have been analyzed. The accuracy of both the basis sets and the CPPs is assessed through benchmark calculations at the coupled-cluster level of theory for atomic and molecular properties. Agreement with all-electron results is much improved relative to the basis sets that originally accompanied the ccECPs; moreover, the combination of cc-pV(*n*+d)Z-ccECP and CPPs is found to be a computationally efficient and accurate alternative to including core electrons in the correlation treatment.

## Introduction

The use of ab initio quantum chemistry methods to investigate the properties, thermochemistry, and reactivity of molecules relies on the expansion of the wave function in products of one-electron orbitals, which are typically expressed in the basis of a linear combination of Gaussian-type functions. The choice of this basis set dictates both the accuracy and computational efficiency of quantum chemical calculations and has been the subject of a number of reviews.^1−3^ The correlation consistent (cc) basis sets, which are the focus of the current work, were originally developed by Dunning to systematically approach the complete basis set (CBS) limit.^4^ A large body of work over the last three decades has resulted in cc basis sets available for almost all of the elements in the periodic table, with consistency in the exponents being energy optimized and using a general contraction scheme. They are typically denoted cc-pV*n*Z (correlation consistent polarized valence *n*-zeta) basis sets, where *n* = D, T, Q, 5, ..., and are designed in a modular fashion. This allows for the addition of functions to address common problems. For example, augmenting with diffuse functions (denoted aug-cc-pV*n*Z) gives a better description of anions, produces significantly better results for electron affinities of atoms, and is important in calculating molecular properties such as polarizabilities and intermolecular interactions.^5^

Correlation consistent basis sets for the second-row elements Al–Ar were originally published in 1993.^6^ However, a later investigation by Bauschlicher and Partridge reported that the aug-cc-pV*n*Z basis sets produced unacceptably large errors for the atomization energy of SO_2_.^7^ Careful evaluation of the performance of these basis sets showed that the addition of large-exponent (tight)-d functions led to major improvements in these benchmarks. Further analysis by Martin revealed that tight-d functions can also have a large effect on the Hartree–Fock energies of molecules containing second-row elements in a high oxidation state, with the involvement of 3d functions in the bonding orbitals, and a suggested term of “inner polarization functions” for basis functions of this type.^8,9^ As a result of the highlighted basis set deficiencies, a new generation of cc-pV(*n*+d)Z basis sets were developed by Dunning et al.^10^ It has been recommended that for second-row p-block elements only these newer “plus d” sets should be used because the minor increase in the total number of basis functions is typically offset by the increased accuracy. However, with the desire to perform electronic structure calculations on ever larger molecules and the rise of high-throughput and data-based approaches, there are substantial benefits to minimizing the number of basis functions while still retaining acceptable computational accuracy.

Effective core potentials (ECPs) reduce the computational effort of a given calculation relative to an equivalent all-electron treatment. They achieve this by separating the core and valence electrons, an idea common throughout chemistry. The most popular ECPs within molecular quantum chemistry follow the pseudopotential (PP) approach, and we make no distinction between ECPs and PPs herein. Instead, the interested reader is directed toward the detailed review of Dolg and Cao.^11^ Recent developments in the calculation of integrals over ECPs have further reduced computational cost, making their use even more attractive.^12−14^ While they are often used to incorporate scalar-relativistic effects for heavier elements without the need for relativistic Hamiltonians, ECPs also offer a partial solution to the problem of large basis sets. Replacing the core electrons with a potential field removes the need for basis functions to describe these electrons. Hence, this reduces the overall size of the basis set. However, the accompanying basis set will need to be specifically paired to a given ECP, increasing the development work required. Within the cc family of basis sets, those paired to small-core ECPs are denoted cc-pV*n*Z-PP and have been developed for a number of heavier elements, including transition metals,^15,16^ alkali metals and alkaline earths,^17^ and some of the actinides.^18^ Where lighter elements of the periodic table are concerned, correlation consistent basis sets for H and B–Ne, denoted cc-pV*n*Z-CDF, have been developed for use with the CASINO Dirac–Fock average relativistic pseudopotentials. However, these sets are intended for applications in quantum Monte Carlo calculations.^19^

Bennett et al. have recently developed a new generation of correlation consistent effective core potentials (ccECPs) for first- and second-row atoms.^20,21^ These are designed specifically for use in correlated electronic structure methods while retaining transferability between atoms and bonded molecules. They use a many-body approach to the construction of the ECPs to circumvent the poor performance for many-body theories seen with ECPs generated in an effective one-particle setting.^22^ To ensure that these new ccECPs could be used in standard electronic structure packages, they chose a commonly used and well established form of the ECP:^23,24^
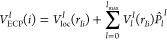
1where  is the effective core potential that supplements the electronic Hamiltonian, with *i* indexing the electrons, *I* the nuclei, and *r*_*Ii*_ the radial distance of electron *i* from the origin of nucleus *I*. This potential is angular momentum dependent with  accounting for core–valence repulsion and  accounting for core–valence orthogonality. Here,  is a projection operator defined as
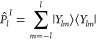
2To ensure orthogonality, *l*_max_ in eq 1 should be equal to the highest angular momentum present in the core.

To be used in quantum chemical calculations, these ECPs require specific basis sets that have been optimized for the valence electrons while using the ECP. Thus, basis sets, denoted ccECP-*n*Z (*n* = D–5) herein, were developed in the same work alongside the new ccECPs (large, neon-core) by minimizing the CCSD(T) ground state atomic energies using an even-tempered progression of exponents.^21^ In addition to optimizing all exponents at the CCSD(T) level, the ccECP-*n*Z basis sets include a number of design elements that differ from the established cc methodology. For example, the same set of s- and p-type primitives and contractions was used across all zeta-levels, which is not seen in the current generation of cc basis sets. More significantly, for the second-row elements Al–Ar, the additional tight-d functions demonstrated to be vital for accurate results are not included, and the sets fail to sufficiently capture the nature of the existing all-electron cc basis sets as the s- and p-type functions do not follow a systematic convergence toward the CBS limit. Despite this, the ccECP-*n*Z construction produces accurate results for excitation energies to low-lying electronic states and equilibrium bond lengths of several diatomic molecules. Short polar bonds, such as those in AlO or SiO, tend to be overbound by ccECP-*n*Z. However, this appears to be relatively common across a number of Ne-core pseudopotentials.^21^ As the ccECPs hold the promise of accurate results at a reduced computational cost, the need for new correlation consistent basis sets paired to the ccECPs for the elements Al–Ar is clear, with potential applications in the computation of extended potential energy surfaces and quantum Monte Carlo or ab initio molecular dynamics simulations.

The same assumption that motivates the use of ECPs in quantum chemistry, namely the separation of core and valence electrons, also places limitations on the ultimate accuracy of correlated wave function methods. The so-called frozen-core approximation, where only the valence electrons enter the correlation treatment, neglects intershell correlation effects to reduce computational cost but relies on said effects being negligible. The effect of core–valence correlation on molecular properties was first studied systematically by Meyer and Rosmus in 1975.^25^ Their work showed that core–valence effects could be nearly as important as valence–correlation effects for alkali metal and alkaline earth compounds. Many subsequent investigations have demonstrated that even for main group elements the core electrons must be correlated in, for example, high-accuracy thermochemistry.^26−29^

In addition to including the correlation of more electron pairs, accurately capturing the core–valence effect requires larger basis sets that have been augmented with tight functions, such as the cc-pCV*n*Z correlation consistent sets.^30^ Optimizing additional functions on the energy difference between correlating all electrons and only correlating valence electrons addresses both the intrashell (core–core) and intershell (core–valence) correlation effects. Subsequent analysis and benchmarking have found that biasing the optimization toward core–valence correlation, known as weighted core–valence or cc-pwCV*n*Z, results in basis sets that converge more rapidly toward the CBS limit for core correlation.^31^ We note that for the second-row elements it is common practice to exclude the low-energy 1s electrons from the correlation treatment, even in “core–valence” calculations. Indeed, the cc-pCV*n*Z and cc-pwCV*n*Z basis sets have been optimized under this assumption.

The physical origin of this core–valence correlation effect is principally the dynamic polarization of the atomic cores by the valence electrons.^32^ This means that these effects, along with static polarization of the cores in the molecular environment, can be accounted for with a core polarization potential (CPP). The development and history of the CPP approach have been reviewed by Dolg and Cao,^11^ based on the pioneering work of Meyer and co-workers^33−35^ and Fuentealba and co-workers.^36^ Briefly, the interaction between a valence electron and the core, λ, is proportional to α_λ_, the core dipole polarizability, leading to
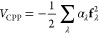
3where **f**_λ_ is the electric field generated at a core by all other cores and the valence electrons, *i*. This electric field is given by
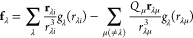
4where a cutoff function, g_λ_(*r*), has been introduced to limit the field to the core region:
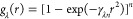
5The parameter γ is fitted to suitable reference data. Two common forms of the cutoff function are used: one is the Fuentealba/Stoll form where *n* = 1,^36^ and the other is the Müller/Meyer form where *n* = 2.^33^ The value of γ is dependent on the functional form chosen.

There have been a small number of investigations where CPPs have been used in conjunction with an all-electron model.^37−40^ Perhaps, the most notable work was by Nicklass and Peterson,^41^ where it was demonstrated that the core–valence effect on the spectroscopic constants of first-row diatomic molecules can be accurately reproduced with a CPP. However, there has been considerably more interest in using CPPs alongside the ECP approximation, where the core electrons have been removed from the system. This combination promises the attractive proposition of accurate and efficient calculations that take into account core–valence correlation effects, without having to add large numbers of additional functions to the basis sets or significantly increase the number of correlated electrons.

The goal of the present work is to develop new correlation-consistent basis sets for the second-row elements Al–Ar specifically matched to the ccECPs of Bennett et al.^21^ The resulting basis sets, denoted (aug-)cc-pV(*n*+d)Z-ccECP (*n* = D, T, and Q), follow the established cc basis set design principles and include the tight-d functions required for accurate properties of molecules containing second-row elements. We note that only basis sets up to quadruple-ζ have been developed, as the CPP code in Molpro does not support orbital angular momentum shells above g.^42,43^ Benchmark calculations on several homonuclear and heteronuclear diatomic molecules are presented to validate the performance of these ECP-based basis sets relative to existing all-electron basis sets. New CPP parameters for Al–Ar have also been optimized and benchmark calculations carried out to demonstrate their efficacy in the computation of core–valence correlation effects.

## Computational Details

All electronic structure calculations in this work were carried out in the Molpro^42,43^ package of programs. The BFGS or simplex algorithms^44^ were used for parameter optimization during basis set development. For the primitive Hartree–Fock (HF) sets, exponents were optimized in symmetry-equivalenced HF calculations, whereby contraction coefficients were extracted from Molpro following the general contraction method of Raffenetti.^45^ For all correlating exponents, optimizations were carried out at the coupled-cluster with single- and double-excitations (CCSD) level. For open-shell species, the Molpro implementation of UCCSD methods, which are spin-unrestricted in the CCSD calculations but use restricted open-shell HF (ROHF) orbitals, was used.

All benchmarking calculations on the new ccECP basis sets, denoted (aug-)cc-pV(*n*+d)Z-ccECP, were carried out at the coupled-cluster with single-, double-, and perturbative triple-excitation [CCSD(T)]^46^ level and were compared to equivalent all-electron calculations. All atomic correlated calculations used symmetry-equivalenced HF reference orbitals, and electron affinities were calculated using diffuse-augmented basis sets. Atomistic benchmarks of the ionization energy were calculated by subtracting the total energy of the neutral atom from the total energy of the cation, while electron affinities were calculated by subtracting the total energy of the anion from the total energy of the neutral atom. For diatomic molecules the equilibrium bond length (*R*_e_), harmonic frequency (ω_e_), and dissociation energy (*D*_e_) were calculated from a seven-point polynomial fit (Dunham analysis).^47^

## Methods

The initial basis set development of the present work closely follows that of the cc-pV*n*Z sets for Al–Ar by Woon and Dunning,^6^ albeit with the ccECP replacing the Ne core. Briefly, Hartree–Fock-optimized primitives are developed for double-, triple-, and quadruple-ζ basis sets. Following this, correlation-consistent polarization functions are determined and added to the HF primitives. Additional tight-d correlating functions^10^ were also optimized, and diffuse-augmented functions were obtained for the lowest energy states of the anions. Initially, a full family of (aug-)cc-pV(*n*+d)Z-ccECP basis sets (where *n* = D, T, and Q) were developed for sulfur, which were subsequently used as guidelines for the rest of the row.

### Hartree–Fock Primitive Sets

The largest difference between the ECP-based basis sets of this work and the all-electron cc-pV*n*Z sets is a decrease in the number of primitive s and p functions due to the removal of the core electrons. As the ccECPs selected for this work define a large Ne-core, the primitive sets can be significantly reduced from the (12s8p), (15s9p), and (16s11p) of the analogous DZ, TZ, and QZ all-electron sets. However, the principles dictating the choice of the primitive set remain the same: a systematic decrease in the basis set incompleteness error in atomic HF calculations and maintaining the qualitative nature of the outermost exponents. The resulting primitive set sizes of (6s5p), (8s7p), and (9s8p) smoothly converge toward the HF/CBS limit. The HF primitive sets were then generally contracted to [1s1p] using atomic orbital coefficients from symmetry-equivalenced HF calculations on the electronic ground states of the atoms.

### Correlating Functions

The number of correlating functions to add to the contracted primitive sets was determined by following the familiar cc approach of using an even-tempered expansion to investigate the incremental lowering of the correlation energy. The resulting cc groupings of functions match those of the analogous all-electron sets and are depicted for sulfur in Figure S1 in the Supporting Information. The even-tempered exponents were subsequently used as starting points for unconstrained optimization of a 1d function for the DZ basis set, 2d1f functions for the TZ basis set, and 3d2f1g functions for the QZ basis set.

**Figure 1:**
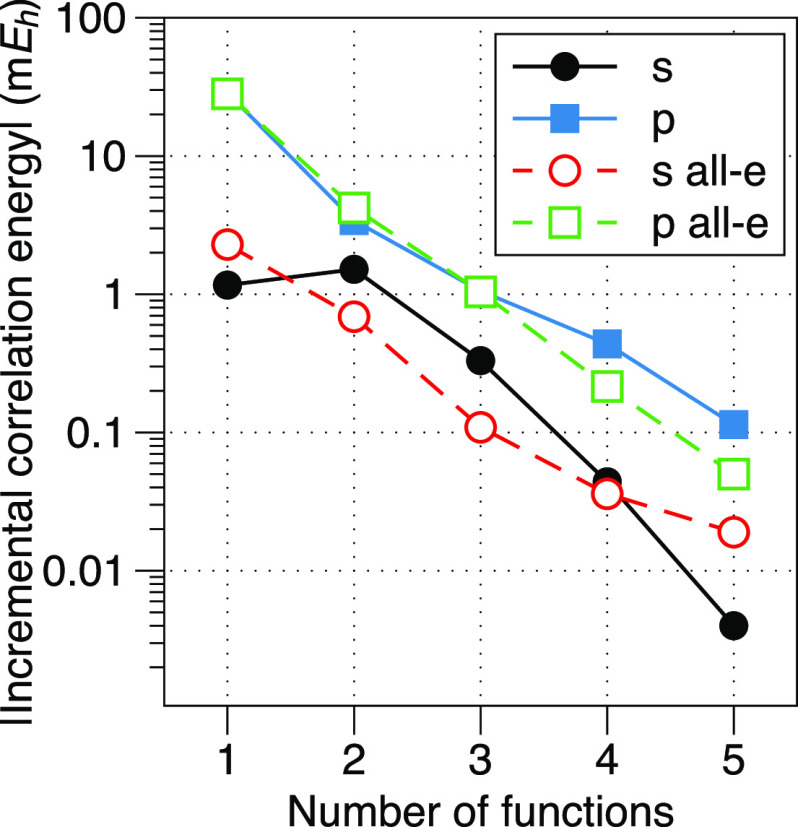
Contribution of s and p correlating functions to the UCCSD correlation energy for the electronic ground state of the S atom. All-electron (all-e) results use the [3s2p]+(3d2f1g) functions from the cc-pVQZ basis set as a base.

The work of Blaudeau et al., Christiansen, and Peterson has indicated that single s-type primitives are poor correlating functions in basis sets designed for use with ECPs.^48−50^ To establish whether this also applies to ccECPs and whether it also affects p-type angular momentum functions for the second-row elements, the correlation energy for sulfur obtained at the UCCSD level by the addition of successive s and p functions is shown in Figure 1. These functions were added to the contracted QZ HF primitives developed above, along with the QZ higher angular momentum correlating functions, to form a [1s1p]+(3d2f1g) base. The results from analogous all-electron calculations, using the [3s2p]+(3d2f1g) taken from the cc-pVQZ set, are also shown.

Focusing initially on p-type functions, it can be seen that both the ECP-based and all-electron functions produce a smooth decrease in the incremental correlation energy as successive functions are added and that the correlation energy recovered is similar for both cases. In contrast, for s-type functions the second ECP-based function recovers a larger amount of correlation energy than the first. After this, subsequent functions proceed to smoothly decrease the incremental correlation energy. This is even more striking when comparison is made to the all-electron case as the first s-type all-electron function recovers roughly twice as much correlation energy as the ECP-based equivalent. An analysis of the exponents indicates that the first ECP-based function has a relatively diffuse exponent, confirming the work of Christiansen.^49^ Given Figure 1, it would appear logical to include (2s1p) correlating functions for a DZ basis set, (3s2p) for TZ, and (4s3p) for QZ. However, as initial testing, shown in Table S1 of the Supporting Information, demonstrated that the inclusion of the additional s-type correlating functions tends to cancel for relative energies (making a negligible difference in the resulting spectroscopic constants), the decision was taken to retain the standard cc groupings of s and p correlating functions of (1s1p), (2s2p), and (3s3p) for DZ–QZ, respectively. This keeps the number of contracted functions as small as possible. As is common practice for cc basis sets, the final s and p correlating functions were uncontracted from the HF sets.

### Additional Tight-d Functions

An additional tight-d function was added to each of the DZ, TZ, and QZ basis sets to avoid the problems previously noted in studies of molecules containing second-row elements.^7−9^ For TZ and QZ, the exponents of the d functions were fixed, and a tighter exponent was optimized at the (U)CCSD level. The tight-d function was then fixed, and the remaining d-type exponents were allowed to relax in a subsequent (U)CCSD optimization. For the DZ set, the new tight-d exponent was determined through a scaling of the TZ exponent by a ratio of ζ_2_(TZ)/ζ_3_(QZ), following ref (10).

The resulting compositions of the cc-pV(*n*+d)Z-ccECP sets are shown in Table 1, along with the analogous ccECP-*n*Z and all-electron cc-pV(*n*+d)Z sets. It can be seen that the basis sets developed in this work have significantly fewer primitive functions than either of the alternatives and, as one would expect, fewer contracted functions than the all-electron cc-pV(*n*+d)Z. This comparison also highlights the lack of tight-d functions in the ccECP-*n*Z sets.

**Table 1:** Composition of the Valence Correlating ccECP-Based Correlation Consistent Basis Sets Developed in This Work for Al–Ar^a^

basis set	composition
cc-pV(D+d)Z-ccECP	(6s5p2d)/[2s2p2d]
cc-pV(T+d)Z-ccECP	(8s7p3d1f)/[3s3p3d1f]
cc-pV(Q+d)Z-ccECP	(9s8p4d2f1g)/[4s4p4d2f1g]
ccECP-DZ	(11s11p1d)/[2s2p1d]
ccECP-TZ	(12s12p2d1f)/[3s3p2d1f]
ccECP-QZ	(13s13p3d2f1g)/[4s4p3d2f1g]
cc-pV(D+d)Z	(12s8p2d)/[4s3p2d]
cc-pV(T+d)Z	(15s9p3d1f)/[5s4p3d1f]
cc-pV(Q+d)Z	(16s11p4d2f1g)/[6s5p4d2f1g]

a The ccECP-*n*Z^21^ and all-electron cc-pV(*n*+d)Z^10^ sets are shown for comparison.

### Diffuse Augmenting Functions

To improve the results for calculations on anions, electron affinities, and polarizabilities, additional diffuse functions were optimized for each basis set to produce aug-cc-pV(*n*+d)Z-ccECP sets. An additional function was added to each angular momentum shell present in the standard basis, and the exponents were energy-optimized for the anion of the respective element, such as the ^2^P_u_ state of the sulfur anion. The tight-d functions were excluded from the basis set for this optimization in keeping with standard methods. Diffuse s and p functions were optimized at the Hartree–Fock level, while higher angular momentum polarization functions were optimized at the (U)CCSD level.

### Core Polarization Potentials

The adjustment of the CPPs follows the method outlined by Nicklass and Peterson,^41^ although a 1s2s2p core was chosen during the adjustment to reflect the heavier elements of the current work. A second notable deviation from the earlier work is that while Nicklass and Peterson adjusted their B–F CPPs to reproduce experimental ionization energies, we have chosen to reproduce CCSD/CBS limit estimates of the first ionization energy. More specifically, we aim to reproduce the core–valence effect on the ionization energy . This is computed as , where  is the ionization energy assembled from the HF energies, the core–valence correlation energies, and the valence–valence correlation energy (that is, excluding the core–core correlation energy that cannot be recovered using a CPP). The  term is constructed from the HF energies and the valence–valence correlation energy in the usual way. Each of these energetic terms is extrapolated to the CBS limit using the Karton–Martin extrapolation formula for HF energies^51^ and the formula of Helgaker and co-workers for correlation energies.^52,53^ All extrapolations used cc-pCV5Z and cc-pCV6Z basis set results.^31,54^

The γ parameters (see eq 5) were subsequently adjusted to reproduce  for Al–Cl based on the (U)CCSD/cc-pV(Q+d)Z valence-only ionization energies. Separate γ parameters were determined for both the *n* = 1 and *n* = 2 forms of the cutoff function. The values of these parameters are given in Table 2, along with the core dipole polarizabilities, α, reproduced from Johnson et al.^55^ It can be seen that the γ values for the *n* = 2 cutoff function are significantly larger than *n* = 1, which is consistent with the observations of Nicklass and Peterson for B–F.^41^

**Table 2:** Core Polarization Potential Cutoff Parameters (γ, See Eq 5) for the Atoms Al–Cl, Adjusted for Both the Fuentealba/Stoll (*n* = 1) and the Müller/Meyer (*n* = 2) Forms of the Cutoff Function^a^

	γ_(*n*=1)_	γ_(*n*=2)_	α
Al	1.5324	4.7998	0.2649
Si	1.7544	5.4614	0.1624
P	1.9926	6.1635	0.1057
S	2.5742	7.8453	0.07205
Cl	2.7965	8.5214	0.05093

a Also presented are the core dipole polarizabilities (α) with respect to the neon isoelectronic series, obtained from ref (55).

**Figure 2:**
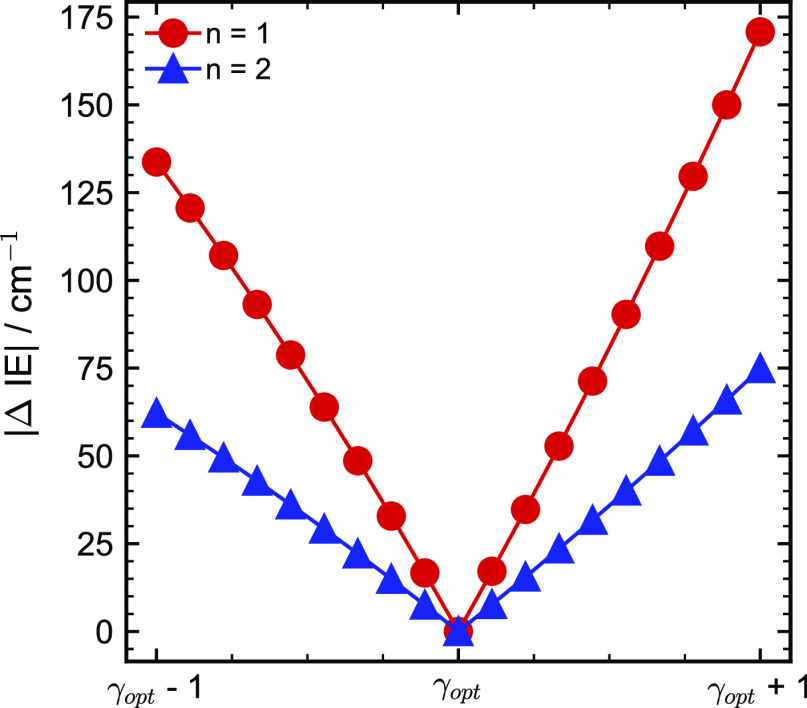
Absolute change in ionization energy (|ΔIE|) for the first ionization energy of sulfur at the (U)CCSD/cc-pV(Q+d)Z/CPP level, where the value of the cutoff parameter (γ) is varied from γ_opt_ – 1 to γ_opt_ + 1. |ΔIE| is plotted for both the Fuentealba/Stoll (*n* = 1) and Müller/Meyer (*n* = 2) forms of the cutoff function.

To identify which form of the cutoff function to use, we quantified the sensitivity of the (U)CCSD/cc-pV(Q+d)Z/CPP first ionization energy of sulfur to the values of γ through the absolute change in ionization energy (|ΔIE|) as γ is varied from γ_opt_ – 1 to γ_opt_ + 1, where γ_opt_ are the optimized values of Table 2. The resulting plot in Figure 2 clearly demonstrates that the Müller/Meyer form (*n* = 2) of the cutoff function is much less sensitive to the value of γ; thus, it is preferred herein.

## Results and Discussion

### Basis Set Benchmarks

#### Atomistic Benchmarks

For the elements Al–Cl, ionization energies and electron affinities have been calculated using the basis sets developed in this work and subsequently compared with those calculated using the cc-pV(*n*+d)Z sets of Dunning and co-workers and the PP-based ccECP-*n*Z basis sets of Bennett et al.^21^ The calculated ionization energies are presented in Table S2 in the Supporting Information, where it can be seen that all three basis set families perform approximately equally. The agreement with experiment is good, and the error introduced by the use of an ECP appears to be minimal.

Table 3 shows the electron affinities for Al–Cl. These have all been calculated using basis sets augmented with additional diffuse functions as it is well-known that such functions are necessary for the correct description of anions.^5^ From Table 3, it is clear that the aug-cc-pV(*n*+d)Z-ccECP basis sets lead to smaller values of the electron affinities than the corresponding aug-cc-pV(*n*+d)Z basis sets, although there is relatively good agreement throughout. This effect becomes smaller as the basis set size is increased, with a mean average deviation of −1.04 kcal mol^–1^ at the DZ level, −0.44 kcal mol^–1^ at the TZ level, and −0.32 kcal mol^–1^ at the QZ level. The convergence of the aug-cc-pV(*n*+d)Z-ccECP results with the basis set is generally smooth. However, there is a more significant DZ to TZ increment for P than for any of the other elements, similar to what is observed with the all-electron aug-cc-pV(*n*+d)Z sets. Experimental electron affinities are presented in Table 3 to provide context for the CCSD(T) values calculated in this work. However, we note there are significant post-CCSD(T) effects,^60^ including scalar relativistic and spin–orbit splitting, that we do not include in the calculated values of Table 3. The all-electron aug-cc-pV(*n*+d)Z results should instead be considered as the “ground-truth” values that the ECP-based sets aim to reproduce.

**Table 3:** Electron Affinities (kcal mol^–1^) at the CCSD(T) Level of Theory for the Atoms Al–Cl

family	*n*Z	Al	Si	P	S	Cl
aug-cc-pV(*n*+d)Z-ccECP	DZ	7.71	28.95	6.63	39.78	78.07
	TZ	9.64	31.84	13.43	44.33	80.51
	QZ	9.94	32.34	15.30	46.56	83.03
aug-ccECP-*n*Z	DZ	4.09	24.11	– 6.12	27.55	65.47
	TZ	9.63	31.88	13.39	44.36	80.51
	QZ	9.03	32.32	12.89	43.89	80.44
aug-cc-pV(*n*+d)Z	DZ	8.42	29.72	8.15	41.04	79.01
	TZ	9.84	32.07	14.17	44.97	80.92
	QZ	10.09	32.45	15.90	47.02	83.29
experiment^56−59^		9.98	32.04	17.22	47.90	83.31

Comparing the newly developed aug-cc-pV(*n*+d)Z-ccECP with the aug-ccECP-*n*Z sets that use the same ECPs, it can be seen that the new sets offer a significant improvement at both the DZ and QZ levels, relative to the all-electron results. Indeed, the mean average deviation between aug-ccECP-*n*Z and aug-cc-pV(*n*+d)Z of −10.24 kcal mol^–1^ at the DZ level, −0.44 kcal mol^–1^ at the TZ level, and −2.04 kcal mol^–1^ at the QZ level highlights significant convergence problems with the aug-ccECP-*n*Z sets as the TZ results are closer to the limiting value than the QZ results. It should also be noted that the electron affinity of P with CCSD(T)/aug-ccECP-DZ is −6.12 kcal mol^–1^, with the neutral atom predicted to be lower in energy than the anion. Further analysis indicates that this is due to the augmenting “diffuse” p exponent of Bennett et al. being too tight; replacing this with the analogous diffuse exponent from aug-cc-pVDZ results in an electron affinity of the correct sign (+6.55 kcal mol^–1^).

#### Diatomic Molecule Benchmarks

Dissociation energies, equilibrium bond lengths, and harmonic frequencies were calculated for the homonuclear diatomic molecules Al_2_–Cl_2_ as well as for sulfur oxide (SO) using the basis sets developed in this work, as well as with the cc-pV(*n*+d)Z and ccECP-*n*Z sets for comparison purposes. These spectroscopic constants were calculated through a seven-point polynomial fit (Dunham analysis^47^) for the ground state of each dimer. The ground state of Al_2_ is ^3^Π_u_, so the CCSD(T) calculation used symmetry-equivalenced reference orbitals.^61^

Table 4 shows the dissociation energies for all six molecules, where it can be seen that the new ECP-based sets lead to smaller values of the dissociation energy, compared to the equivalent all-electron cc-pV*n*Z basis, at the DZ and QZ level but a higher value at the TZ level. However, the absolute difference is typically within 1 kcal mol^–1^ (except for P_2_/cc-pV(D+d)Z-ccECP, where it is 1.24 kcal mol^–1^), and the mean average deviations are −0.55, +0.48, and −0.27 kcal mol^–1^ at the DZ, TZ, and QZ level, respectively. The convergence with the basis set is smooth and follows the general trend of the all-electron sets. In contrast, the mean average deviation between ccECP-*n*Z and cc-pV(*n*+d)Z sets is 10.45 kcal mol^–1^ at the DZ level, 2.28 kcal mol^–1^ at the TZ level, and 0.91 kcal mol^–1^ at the QZ level. Comparing the mean average deviations of the new cc-pV(*n*+d)Z-ccECP sets with the ccECP-*n*Z sets shows that there are significant improvements for all basis set qualities.

**Table 4:** Dissociation Energies (kcal mol^–1^) at the CCSD(T) Level of Theory for the Diatomic Molecules Al_2_–Cl_2_ and SO

family	*n*Z	Al_2_	Si_2_	P_2_	S_2_	Cl_2_	SO
cc-pV(*n*+d)Z-ccECP	DZ	27.92	61.58	91.15	85.21	43.69	100.05
	TZ	31.89	71.28	105.63	96.46	53.70	118.35
	QZ	32.65	73.92	111.09	99.91	56.51	122.39
ccECP-*n*Z	DZ	24.65	52.29	75.81	71.32	36.38	89.73
	TZ	31.08	69.16	102.47	92.53	50.84	114.68
	QZ	32.48	73.36	110.41	98.91	55.55	121.53
cc-pV(*n*+d)Z	DZ	28.33	62.33	92.39	85.33	43.76	100.74
	TZ	31.75	70.98	105.37	95.47	53.51	117.35
	QZ	32.67	74.00	111.67	100.13	57.04	122.20
experiment^61−63^		31.70	75.60	117.20	102.90	59.70	126 ± 1

Calculated equilibrium bond lengths for all six diatomic molecules are presented in Table 5. Comparing the newly developed sets to the all-electron results again shows that the use of an ECP leads to shorter bond lengths. This effect is greatest for the TZ basis sets, as shown by the mean average deviation across all six dimers: – 0.0030 Å at the DZ level, −0.0102 Å at the TZ level, and −0.0054 Å at the QZ level. Comparing the dimers themselves, the deviation is larger for the two lighter atom pairs, Al_2_ and Si_2_. If the mean average deviation is calculated for only these two diatomic molecules, then the results are −0.0057 Å for the DZ level, −0.0162 Å for the TZ level, and −0.0088 Å for the QZ level. The best agreement occurs for SO, with values of −0.0000, −0.0016, and −0.0024 Å for DZ, TZ, and QZ calculations, respectively. Generally, the new basis sets converge in a similar manner to the all-electron sets.

**Table 5:** Equilibrium Bond Lengths (Å) at the CCSD(T) Level of Theory for the Diatomic Molecules Al_2_–Cl_2_ and SO

family	*n*Z	Al_2_	Si_2_	P_2_	S_2_	Cl_2_	SO
cc-pV(*n*+d)Z-ccECP	DZ	2.7464	2.2787	1.9222	1.9169	2.0266	1.5150
	TZ	2.7028	2.2494	1.9021	1.8983	1.9959	1.4900
	QZ	2.7043	2.2462	1.8968	1.8929	1.9934	1.4838
ccECP-*n*Z	DZ	2.8410	2.3644	1.9833	1.9887	2.0999	1.5527
	TZ	2.7223	2.2640	1.9115	1.9117	2.0157	1.4976
	QZ	2.7087	2.2497	1.8980	1.8957	1.9964	1.4855
cc-pV(*n*+d)Z	DZ	2.7472	2.2831	1.9247	1.9189	2.0350	1.5150
	TZ	2.7220	2.2625	1.9097	1.9057	2.0079	1.4916
	QZ	2.7139	2.2542	1.9019	1.8969	1.9966	1.4862
experiment^61−63^		2.701	2.246	1.8934	1.8892	1.9879	1.4811

As mentioned previously, Ne-core ECPs are known to produce shorter bond lengths than all-electron calculations,^21^ which is consistent with the results of Table 5. The SO equilibrium bond length having the smallest deviation from all-electron results adds extra evidence to this: the oxygen atom does not use an ECP; thus, the overbinding effect is smaller. This may also explain why the lighter second-row elements have the greatest overbinding, as a larger proportion of the electrons are replaced by an ECP. Table 5 also shows that the ccECP-*n*Z sets produce bond lengths that are systematically too short, as the size of the basis tends toward the limit. Conversely, at the DZ and TZ levels the bonds lengths are too long relative to the all-electron calculations, leading to poor convergence with basis set size.

Table 6 shows the calculated harmonic frequencies for all six molecules across the three basis set families. Comparing the cc-pV(*n*+d)Z-ccECP sets with the cc-pV(*n*+d)Z sets reveals good agreement throughout, with better agreement as the zeta-level increases. The absolute mean average deviation is 3.7 cm^–1^ at the DZ level, 3.3 cm^–1^ at the TZ level, and 0.8 cm^–1^ at the QZ level. Convergence with the basis set size is generally smooth, except for Al_2_, which has a larger harmonic frequency at the TZ level than the QZ level. However, this is consistent with the all-electron calculations. Comparing the new cc-pV(*n*+d)Z-ccECP sets with the ccECP-*n*Z sets, it can be seen that the new basis sets offer significant improvements across the board, with large improvements observed with small basis sets. This is particularly striking for ccECP-DZ, which has a mean average deviation, relative to the all-electron calculation, of −40.4 cm^–1^.

**Table 6:** Harmonic Frequencies (cm^–1^) at the CCSD(T) Level of Theory for the Diatomic Molecules Al_2_–Cl_2_ and SO

family	*n*Z	Al_2_	Si_2_	P_2_	S_2_	Cl_2_	SO
cc-pV(*n*+d)Z-ccECP	DZ	279.3	498.0	762.3	712.7	519.1	1077.5
	TZ	287.1	513.6	775.0	725.7	552.3	1153.0
	QZ	285.6	515.4	782.3	728.1	555.6	1154.2
ccECP-*n*Z	DZ	263.3	463.4	707.1	659.6	492.6	1023.9
	TZ	281.2	505.2	767.9	711.4	541.9	1138.8
	QZ	284.5	513.1	780.8	725.9	555.0	1151.6
cc-pV(*n*+d)Z	DZ	279.6	497.6	764.6	709.8	512.9	1087.8
	TZ	285.0	511.4	773.5	718.8	549.1	1149.3
	QZ	285.0	514.8	783.2	728.7	557.4	1154.3
experiment^61−63^		285.8	510.98	780.77	725.65	559.70	1149.22

Overall, it can be seen that the (aug-)cc-pV(*n*+d)Z-ccECP basis sets developed in this work produce results that are significantly closer to those from the all-electron (aug-)cc-pV(*n*+d)Z sets than the (aug-)cECP-*n*Z sets that use the same ECPs. This is particularly evident at the DZ level but remains significant even at QZ. The new basis sets converge smoothly with basis set size and in the vast majority of cases agree well with (aug-)cc-pV(*n*+d)Z results at the QZ level, implying that any error introduced by the use of the ECPs is small when combined with appropriate basis sets. However, for the lighter elements, equilibrium bond lengths are underestimated. As cc-pV(*n*+d)Z-ccECP and ccECP-*n*Z appear to tend toward the same limits for bond lengths, this indicates that the error is likely to be related to the use of a large-core ECP.

### CPP Benchmarks

As CPPs account for both core–valence effects and the static polarization of atomic cores by the molecular environment, the comparison of the effect of core–valence correlation (that is, the difference between a given property in valence-only and core–valence correlating calculations) between all-electron and ECP with CPP-based calculations, as usually done with correlation consistent basis sets for core–valence correlation, is not a fair one. Instead, to validate the performance of the combination of the basis sets and CPPs developed in this work, we compared the computed spectroscopic properties of the homonuclear diatomic molecules Al_2_–Cl_2_ computed with the all-electron cc-pCV*n*Z basis sets with those from cc-pV(*n*+d)Z-ccECP/CPP. All calculations were carried out with the CCSD(T) method on the electronic ground state of the molecule, and all-electron calculations used a 1s frozen core. In the cc-pV(*n*+d)Z-ccECP/CPP calculations, all electrons not replaced by the ECP were correlated, and the Müller/Meyer form of the CPP cutoff function was used. In keeping with methods employed by Peterson and Dunning,^31^ atomic calculations carried out for dissociation energies were not performed using symmetry-equivalenced reference orbitals.

Tables 7, 8, and 9 display the calculated dissociation energies, equilibrium bond lengths, and harmonic frequencies, respectively, of the five diatomic molecules. Focusing initially on the dissociation energies in Table 7, it can be seen that the combination of the cc-pV(*n*+d)Z-ccECP basis and CPP reproduces the all-electron cc-pCV*n*Z results well. The agreement between the two approaches increases with basis set size, and fortuitously, the cc-pV(*n*+d)Z-ccECP/CPP results tend to be slightly closer to the basis set limit than the equivalent cc-pCV*n*Z. The dissociation energies computed with the CPP approach also converge smoothly toward the basis set limit. However, as with the all-electron results, there is a large difference between DZ and TZ results.

**Table 7:** Dissociation Energies (kcal mol^–1^) at the CCSD(T) Level of Theory for the Homonuclear Diatomic Molecules Al_2_–Cl_2_, Including Core–Valence Correlation Effects

family	*n*Z	Al_2_	Si_2_	P_2_	S_2_	Cl_2_
cc-pV(*n*+d)Z-ccECP/CPP	DZ	28.61	62.77	92.65	86.53	44.47
	TZ	32.21	72.08	106.92	97.54	54.23
	QZ	32.74	74.49	112.15	100.87	57.01
cc-pCV*n*Z	DZ	28.37	61.82	90.82	83.19	42.76
	TZ	31.66	71.03	105.95	95.76	53.65
	QZ	32.60	74.24	112.54	100.67	57.13
	5Z	32.90	75.22	114.71	102.39	58.53
	6Z	33.02	75.65	115.66	103.14	59.10
experiment^61−63^		31.70	75.60	117.20	102.90	59.70

**Table 8:** Equilibrium Bond Lengths (Å) at the CCSD(T) Level of Theory for the Homonuclear Diatomic Molecules Al_2_–Cl_2_, Including Core–Valence Correlation Effects

family	*n*Z	Al_2_	Si_2_	P_2_	S_2_	Cl_2_
cc-pV(*n*+d)Z-ccECP/CPP	DZ	2.7177	2.2596	1.9095	1.9063	2.0157
	TZ	2.6795	2.2338	1.8909	1.8885	1.9871
	QZ	2.6823	2.2313	1.8862	1.8836	1.9853
cc-pCV*n*Z	DZ	2.7525	2.2909	1.9329	1.9331	2.0465
	TZ	2.7162	2.2580	1.9054	1.9027	2.0045
	QZ	2.7018	2.2458	1.8952	1.8913	1.9914
	5Z	2.6992	2.2431	1.8922	1.8880	1.9874
	6Z	2.6979	2.2422	1.8913	1.8868	1.9859
experiment^61−63^		2.701	2.246	1.8934	1.8892	1.9879

**Table 9:** Harmonic Frequencies (cm^–1^) at the CCSD(T) Level of Theory for the Homonuclear Diatomic Molecules Al_2_–Cl_2_, Including Core–Valence Correlation Effects

family	*n*Z	Al_2_	Si_2_	P_2_	S_2_	Cl_2_
cc-pV(*n*+d)Z-ccECP/CPP	DZ	281.9	502.3	769.3	719.3	526.8
	TZ	289.4	517.4	781.2	732.1	556.3
	QZ	286.0	518.8	788.4	733.7	558.7
cc-pCV*n*Z	DZ	278.7	494.5	759.3	703.4	512.5
	TZ	283.7	511.2	777.0	720.5	551.8
	QZ	286.8	516.9	788.4	732.3	560.0
	5Z	286.0	517.5	790.6	734.6	564.1
	6Z	286.4	518.1	791.9	736.0	565.1
experiment^61−63^		285.8	511.0	780.8	725.7	559.7

The equilibrium bond lengths presented in Table 8 are an interesting set of results. The agreement between the two approaches for any given basis set zeta-level is less than ideal, with the CPP-based approach underestimating the all-electron bond length in all cases. The agreement does increase with zeta-level, and typically the agreement is also better for the heavier elements under consideration, the latter of which is consistent with the trend for valence-only results in Table 5. The consistently too-short bond lengths do lead to some fortuitous error cancellation, with cc-pV(D+d)Z-ccECP/CPP producing bond lengths roughly equivalent to the considerably more expensive cc-pCVTZ results. The cc-pV(T+d)Z-ccECP/CPP lengths are also similar to those of cc-pCV5Z, although by this point the Al_2_ and Si_2_ bonds are already shorter than the cc-pCV6Z results and potentially beyond the all-electron basis set limit. This acts as another reminder that large-core ECPs for these elements can lead to bond lengths that are too short.

Trends similar to those observed for the dissociation energies are also seen for the harmonic frequencies in Table 9. The agreement between the approaches is relatively good and improves with basis set size. Again, the cc-pV(D+d)Z-ccECP/CPP result is better than the cc-pCVDZ result and approaches the cc-pCVTZ result. This extends to other zeta-levels, such that cc-pV(*n*+d)Z-ccECP/CPP gives results roughly equivalent to cc-pCV(*n*+1)Z. Thus, owing to the use of ECP and the lack of core correlating basis functions and because fewer electrons are entering the correlation treatment, a considerable saving of computational effort is achieved.

The savings in computational cost are demonstrated in Tables 10 and 11, which present the CPU times for single-point CCSD(T) energy evaluations on pentathiolane (S_5_). All of the calculations were carried out in *C*_1_ symmetry, and the timings are taken as the mean average of three individual calculations that were all performed on a single core of an Intel i7-8700 CPU with 16 GB of RAM. The exception to this is the cc-pCVQZ calculation in Table 11, where the value is for a single run owing to the very-long runtime. The timings are further broken down into the components of the calculation, such as time spent in integral evaluation, HF, etc., in the Supporting Information. Table 10 compares the time taken for valence-only calculations with the new cc-pV(*n*+d)Z-ccECP sets against cc-pV(*n*+d)Z; hence, it shows any reduction in computational cost from the use of an ECP and the basis sets developed in this work. As percentages relative to the time taken for the analogous cc-pV(*n*+d)Z calculation, cc-pV(*n*+d)Z-ccECP takes 68% of the time at the DZ level, 83% at TZ, and 85% at QZ.

**Table 10:** CPU Times for a Valence-Only Single-Point CCSD(T) Energy Evaluation on Pentathiolane

family	*n*Z	time (s)
cc-pV(*n*+d)Z-ccECP	DZ	26.7
	TZ	288.6
	QZ	2252.0
cc-pV(*n*+d)Z	DZ	39.2
	TZ	348.3
	QZ	2651.8

**Table 11:** CPU Times for a Core–Valence Single-Point CCSD(T) Energy Evaluation on Pentathiolane

family	*n*Z	time (s)
cc-pV(*n*+d)Z-ccECP/CPP	DZ	26.3
	TZ	280.7
	QZ	2179.6
cc-pCV*n*Z	DZ	741.0
	TZ	17388.6
	QZ	200464.9

Table 11 shows that significantly more impressive gains in computational efficiency are observed when a CPP is used for core–valence correlation rather than the conventional approach using a cc-pCV*n*Z basis. Again, as percentages relative to the time taken for the analogous cc-pCV*n*Z calculation (with a 1s frozen core), cc-pV(*n*+d)Z-ccECP/CPP takes 4% at the DZ level, 2% at the TZ level, and 1% at QZ. Crucially, cc-pV(T+d)Z-ccECP/CPP takes less than half the CPU time of the cc-pCVDZ calculation, and cc-pV(Q+d)Z-ccECP/CPP is almost an order of magnitude faster than the lower zeta-level cc-pCVTZ. A comparison of Table 11 with Table 10 indicates that the calculation using the CPP can be marginally faster than the analogous calculation without it. The timing breakdowns in the Supporting Information indicate that this is primarily due to a reduction in the time taken for HF self-consistent field convergence.

## Conclusions

Correlation consistent basis sets for the second-row elements Al–Ar, denoted cc-pV(*n*+d)Z-ccECP, have been developed for use with the large-core correlation consistent ECPs of Bennett et al.^21^ The new basis sets are designed as a replacement for the sets that were provided with these ECPs, ensuring that the resulting basis sets follow the established correlation consistent design philosophy and include the tight-d functions that are known to be important for the second row. The basis sets are accompanied by newly adjusted CPPs to recover the effects of core–valence correlation; moreover, it is found that the *n* = 2 form of the CPP cutoff function is least sensitive to the value of the cutoff parameter.

Benchmarking of the new basis sets at the CCSD(T) level on atomic ionization energies and electron affinities and on the dissociation energy and harmonic frequencies of diatomic molecules demonstrates that the new cc-pV(*n*+d)Z-ccECP sets produce results significantly closer to those from the all-electron cc-pV(*n*+d)Z when compared to the ccECP-*n*Z provided with the ECPs. In fact, the new basis sets reproduce the all-electron benchmark well, and any deviations decrease with basis set size. It also follows that the new cc-pV(*n*+d)Z-ccECP sets converge smoothly toward the basis set limit, as expected for a correlation consistent basis. Analysis of computed equilibrium bond lengths for homonuclear diatomic molecules reveals that they are underestimated with both the cc-pV(*n*+d)Z-ccECP and ccECP-*n*Z sets and that the underestimation increases for the lighter elements. As both of the ECP-based basis sets appear to be converging toward the same limit, it appears that this overbinding is likely due to the ECP rather than the basis set. However, it is unclear whether this could be addressed by further adjustment of the ECPs or that it is unavoidable when using a large-core ECP for the second-row elements.

The CPPs are benchmarked on the spectroscopic properties of homonuclear diatomic molecules and calibrated against the all-electron core–valence cc-pCV*n*Z results. For dissociation energies and harmonic frequencies, the cc-pV(*n*+d)Z-ccECP/CPP approach produces accurate values with minimal computational expense compared to extensive calculations with large numbers of correlated electrons. As with the valence-only cc-pV(*n*+d)Z-ccECP calculations, equilibrium bond lengths are underestimated for the lighter elements. For all of the spectroscopic properties, at a given zeta-level the CPP-based results are slightly closer to the basis set limit than the equivalent cc-pCV*n*Z. This is most pronounced for DZ, where cc-pV(D+d)Z-ccECP/CPP results are roughly comparable to those of cc-pCVTZ. In general, the accuracy and low computational expense of the CPP approach here are a continuation of what was observed by Nicklass and Peterson for B–F^41^ and, in our opinion, are underexplored tools for large molecular systems or high-throughput computation/benchmarking.

## Supporting Information Available

Plot of correlation consistent groupings of angular momentum functions, benchmarking of the effect of an additional correlation s-function on various properties, tabulated ionization energies, and further timing data (PDF)
Basis set parameters in Molpro format (ZIP)
