# Supplementary material for: Correlation Consistent Basis Sets and Core Polarization Potentials for Al–Ar with ccECP Pseudopotentials
Source: J Phys Chem A. 2022 Aug 17;126(34):5853–63. doi: 10.1021/acs.jpca.2c04446 (PMC9442647; doi:10.1021/acs.jpca.2c04446)
Supplement: Supplementary file 1 — jp2c04446_si_001.pdf [file jp2c04446_si_001.pdf]

Supporting Information: Correlation Consistent Basis Sets and Core Polarization Potentials for Al–Ar with ccECP Pseudopotentials

Adam N. Hill, Anthony J. H. M. Meijer, and J. Grant Hill*

Department of Chemistry, University of Sheffield, Sheffield S3 7HF, U.K.

E-mail: grant.hill@sheffield.ac.uk

Correlation consistent groupings

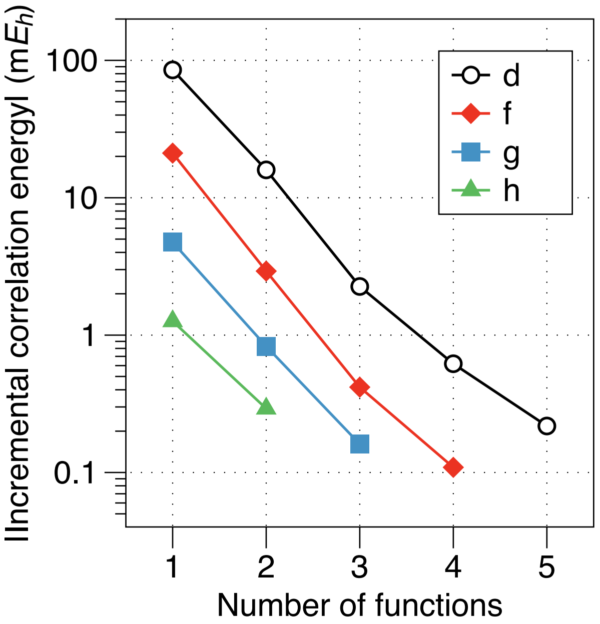

Figure S1: Contribution of d–h angular momentum functions to the UCCSD correlation energy for the electronic ground state of the S atom.

Benchmarking

Extra correlating *s*-function

Table S1: Spectroscopic constants, ionisation energies, and electron affinities of sulfur and aluminium comparing three families of basis sets: the ccECP basis sets developed in this work; those same sets with an extra correlating *s*-function; and the equivalent all electron basis set.

s & *p* Functions		Basis set	DE	R_e	ω_e	IP	EA
**S/S_2**	2s2p	cc-pV(D+d)Z-ccECP	85.21	1.9169	712.69	222.49	39.78
	3s3p	cc-pV(T+d)Z-ccECP	96.46	1.8983	725.66	231.47	44.33
	4s4p	cc-pV(Q+d)Z-ccECP	99.91	1.8929	728.16	235.13	46.56
	3s2p	cc-pV(D+d)Z-ccECP	85.74	1.9158	709.91	221.57	40.70
	4s3p	cc-pV(T+d)Z-ccECP	95.29	1.9011	718.86	231.79	44.48
	5s4p	cc-pV(Q+d)Z-ccECP	99.75	1.8932	727.00	235.18	46.56
		cc-pV(D+d)Z	85.33	1.9189	709.80	222.27	41.04
		cc-pV(T+d)Z	95.47	1.9057	718.80	232.81	44.97
		cc-pV(Q+d)Z	100.13	1.8969	728.69	236.14	47.02
**Al/Al_2**	2s2p	cc-pV(D+d)Z-ccECP	27.92	2.7476	277.56	137.99	7.71
	3s3p	cc-pV(T+d)Z-ccECP	31.89	2.7056	285.66	137.43	9.64
	4s4p	cc-pV(Q+d)Z-ccECP	32.65	2.7041	285.04	137.64	9.94
	3s2p	cc-pV(D+d)Z-ccECP	28.19	2.7484	277.02	135.42	8.30
	4s3p	cc-pV(T+d)Z-ccECP	31.61	2.7065	285.34	137.31	9.68
	5s4p	cc-pV(Q+d)Z-ccECP	32.65	2.7043	285.48	137.64	9.94
		cc-pV(D+d)Z	28.33	2.7472	279.61	134.82	8.42
		cc-pV(T+d)Z	31.75	2.7220	285.01	137.00	9.84
		cc-pV(Q+d)Z	32.67	2.7139	284.96	137.54	10.09

Ionisation energies

Table S2: Ionisation energies (kcal mol^{-1}) at the CCSD(T) level of theory for the atoms Al–Cl.

Basis	Al	Si	P	S	Cl
cc-pV(D+d)Z-ccECP	137.99	186.13	238.85	222.49	285.31
cc-pV(T+d)Z-ccECP	137.43	187.21	241.58	231.47	292.45
cc-pV(Q+d)Z-ccECP	137.64	187.79	242.51	235.13	296.43
ccECP-DZ	138.67	187.37	240.81	225.69	289.72
ccECP-TZ	137.74	187.78	242.26	232.63	293.94
ccECP-QZ	137.65	188.00	242.92	235.85	297.56
cc-pV(D+d)Z	134.82	183.42	236.23	222.27	284.82
cc-pV(T+d)Z	137.00	187.01	241.24	232.81	293.16
cc-pV(Q+d)Z	137.54	187.59	242.13	236.14	296.87
Experiment[S1–S5]	138.04	187.99	241.83	238.91	299.05

Timing data

The CPU timing data presented in Tables S3 and S4 are the components of a single-point CCSD(T) energy evaluation on pentathiolane (S_5) in C_1 symmetry. All timings are the mean average of three individual calculations, performed on a single core of an Intel i7-8700 with 16 GB of RAM. The exception to this is the cc-pCVQZ data in Table S4, where the values are from a single calculation due to the very-long runtime. The timings are also presented visually as stacked bar charts in Figures S2–S4

Table S3: CPU timing breakdown (s) for a valence-only single point CCSD(T) energy evaluation on pentathiolane.

Family	nZ	Integrals	HF	Trans.	CCSD	(T)
cc-pV(n+d)Z-ccECP	DZ	0.6	0.6	0.3	14.2	10.8
	TZ	5.4	7.7	3.6	115.8	155.9
	QZ	57.3	75.9	32.9	722.7	1363.1
cc-pV(n+d)Z	DZ	1.6	1.8	0.7	23.8	11.1
	TZ	10.6	15.0	5.6	161.5	155.4
	QZ	88.5	112.2	44.4	1057.2	1349.4

Table S4: CPU timing breakdown (s) for a core-valence single point CCSD(T) energy evaluation on pentathiolane.

Family	nZ	Integrals	CPP	HF	Trans.	CCSD	(T)
cc-pV(n+d)Z-ccECP/CPP	DZ	0.6	0.2	0.3	0.3	14.0	10.9
	TZ	4.9	1.3	3.6	3.3	111.7	155.9
	QZ	57.8	16.6	35.4	29.9	681.7	1358.2
cc-pCVnZ	DZ	2.5	—	3.3	3.6	387.8	343.8
	TZ	41.4	—	70.5	72.1	5227.2	11977.2
	QZ	548.3	—	999.4	1303.4	47384.5	150229.0

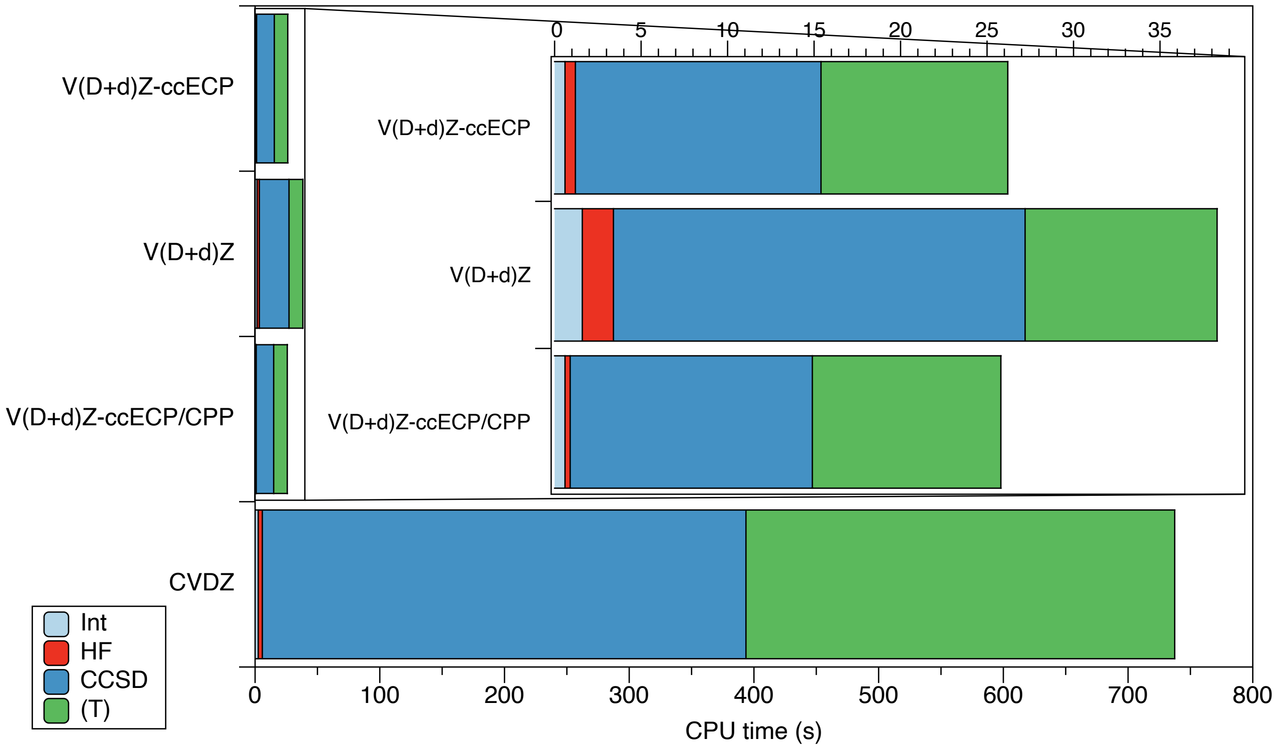

Figure S2: CPU times for single point CCSD(T) double-zeta energy evaluations on pentathiolane. Only the integral evaluation (Int), HF, CCSD and (T) components of the calculation are shown. A zoomed area is used to highlight the timings of the faster calculations.

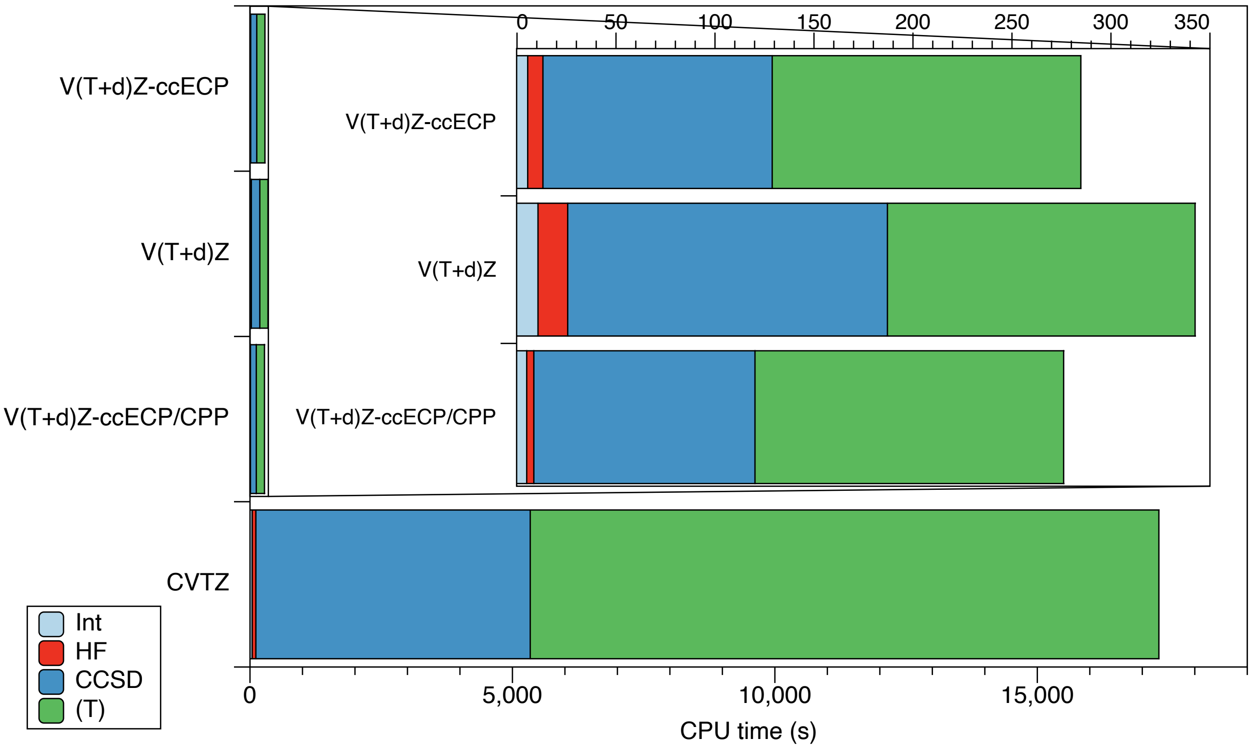

Figure S3: CPU times for single point CCSD(T) triple-zeta energy evaluations on pentathiolane. Only the integral evaluation (Int), HF, CCSD and (T) components of the calculation are shown. A zoomed area is used to highlight the timings of the faster calculations.

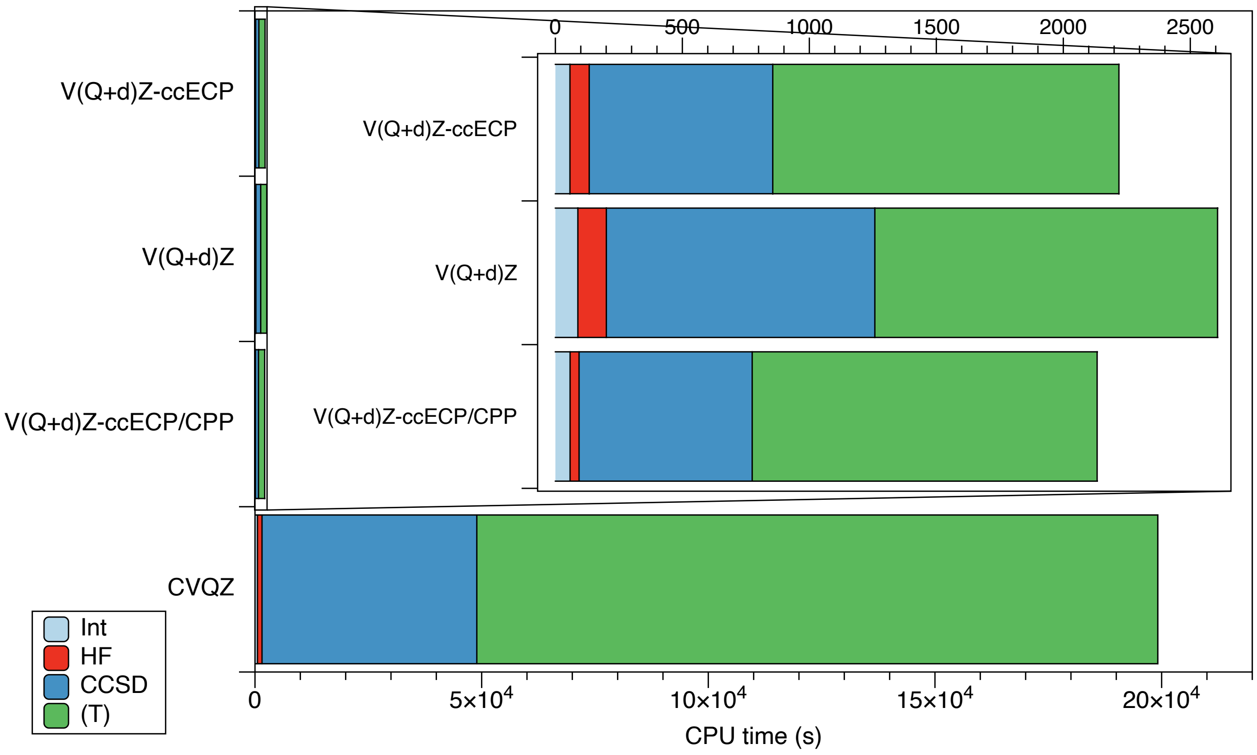

Figure S4: CPU times for single point CCSD(T) quadruple-zeta energy evaluations on pentathiolane. Only the integral evaluation (Int), HF, CCSD and (T) components of the calculation are shown. A zoomed area is used to highlight the timings of the faster calculations.

Basis Sets in Molpro Format

All of the following basis sets must be used in conjunction with the ccECPs of Bennett *et al.*, *J. Chem. Phys.* **149**, 104108 (2018).

cc-pV(D+d)Z-ccECP

```
s,Al, 9.467632E+00, 5.626780E+00, 2.011750E+00, 1.153557E+00, 1.680911E-01, 6.127412E-02
c,1.6, 2.106000E-03, -5.525000E-03, 6.608500E-02, -2.884150E-01, 6.652750E-01, 4.728490E-01
c,6.6, 1.0
p,Al, 5.086488E+00, 3.178563E+00, 1.986886E+00, 1.961017E-01, 5.681130E-02
c,1.5, -5.667000E-03, 1.819200E-02, -2.883900E-02, 4.918900E-01, 6.154460E-01
c,5.5, 1.0
d,Al, 1.308217E+00, 1.832422E-01

s,Si, 1.108664E+01, 6.688456E+00, 2.621138E+00, 1.512618E+00, 2.341472E-01, 8.553995E-02
c,1.6, 2.178000E-03, -5.499000E-03, 8.033500E-02, -3.226790E-01, 6.862160E-01, 4.647370E-01
c,6.6, 1.0
p,Si, 6.598755E+00, 4.126387E+00, 2.581579E+00, 2.886592E-01, 8.597700E-02
c,1.5, -5.591000E-03, 2.022600E-02, -3.687200E-02, 5.193070E-01, 5.869270E-01
c,5.5, 1.0
d,Si, 1.738470E+00, 2.675427E-01

s,P,1.238236E+01,7.485122E+00,3.082440E+00,1.926247E+00,3.056385E-01,1.119803E-01
c,1.6,2.195000E-03,-5.501000E-03,1.094400E-01,-3.638660E-01,6.998080E-01,4.593350E-01
c,6.6,1.0
p,P,8.332862E+00,5.213576E+00,3.265010E+00,3.917816E-01,1.186369E-01
c,1.5,-5.202000E-03,1.989200E-02,-4.200200E-02,5.369650E-01,5.687700E-01
c,5.5,1.0
d,P,2.161064E+00,3.604973E-01
```

```
s,S,1.371939E+01,8.361204E+00,3.780862E+00,2.363702E+00,3.851312E-01,1.408929E-01
c,1.6,2.159000E-03,-6.249000E-03,1.213550E-01,-3.861230E-01,7.155840E-01,4.512290E-01
c,6.6,1.0
p,S,1.075098E+01,6.729523E+00,4.218423E+00,5.045883E-01,1.473090E-01
c,1.5,-4.307000E-03,1.663900E-02,-3.974800E-02,5.441890E-01,5.668270E-01
c,5.5,1.0
d,S,2.624970E+00,4.600632E-01

s,Cl,1.468532E+01,9.081676E+00,4.517516E+00,2.823726E+00,4.726501E-01,1.727413E-01
c,1.6,3.796000E-03,-1.095200E-02,1.279250E-01,-3.953880E-01,7.245830E-01,4.471770E-01
c,6.6,1.0
p,Cl,1.328317E+01,8.318690E+00,5.220721E+00,6.312944E-01,1.821076E-01
c,1.5,-3.718000E-03,1.493300E-02,-3.997300E-02,5.513830E-01,5.617710E-01
c,5.5,1.0
d,Cl,3.195709E+00,5.780413E-01

s,Ar,1.561888E+01,9.399571E+00,5.294684E+00,3.311215E+00,5.648221E-01,2.065069E-01
c,1.6,1.705000E-03,-2.509000E-03,1.186860E-01,-4.024230E-01,7.324230E-01,4.447530E-01
c,6.6,1.0
p,Ar,1.648115E+01,1.031973E+01,6.477514E+00,7.679845E-01,2.212524E-01
c,1.5,-2.576000E-03,1.244000E-02,-3.908600E-02,5.569660E-01,5.563190E-01
c,5.5,1.0
d,Ar,3.640558E+00,7.022656E-01
```

cc-pV(T+d)Z-ccECP

```
s,Al,8.212835E+00,4.555659E+00,2.241051E+00,1.298574E+00,6.706170E-01,2.208360E-01,
        9.829919E-02,4.356435E-02
```

```
c,1.8,2.969000E-03,-1.335600E-02,7.908900E-02,-2.317920E-01,-9.787600E-02,4.101350E-01,
        5.783300E-01,1.869940E-01
c,7.7,1.0
c,8.8,1.0
p,Al,8.744620E+00,5.193068E+00,2.982244E+00,1.387771E+00,2.586180E-01,9.758792E-02,
        3.679630E-02
c,1.7,9.110000E-04,-5.434000E-03,9.921000E-03,-3.063100E-02,2.871910E-01,5.444390E-01,
        3.013670E-01
c,6.6,1.0
c,7.7,1.0
d,Al,1.814977E+00,3.198831E-01,1.079784E-01
f,Al,2.899838E-01

s,Si,9.665766E+00,5.482739E+00,2.775639E+00,1.643690E+00,8.488039E-01,3.097429E-01,
        1.385535E-01,6.089588E-02
c,1.8,2.722000E-03,-1.138100E-02,9.174100E-02,-2.748170E-01,-9.523000E-02,4.248530E-01,
        5.844440E-01,1.850260E-01
c,7.7,1.0
c,8.8,1.0
p,Si,9.981886E+00,6.178782E+00,3.676271E+00,1.953541E+00,3.720789E-01,1.446960E-01,
        5.503535E-02
c,1.7,1.540000E-03,-7.908000E-03,1.617000E-02,-4.200400E-02,3.158970E-01,5.424010E-01,
        2.731420E-01
c,6.6,1.0
c,7.7,1.0
d,Si,2.391269E+00,4.580241E-01,1.552119E-01
f,Si,3.692667E-01

s,P,1.122502E+01,6.369909E+00,3.337544E+00,1.973366E+00,9.581721E-01,4.051031E-01,
```

```
        1.809439E-01,7.953374E-02
c,1.8,2.402000E-03,-9.789000E-03,9.596100E-02,-3.140620E-01,-8.159000E-02,4.443610E-01,
        5.855890E-01,1.805490E-01
c,7.7,1.0
c,8.8,1.0
p,P,1.181951E+01,6.805271E+00,4.141216E+00,2.587294E+00,4.999773E-01,1.978731E-01,
        7.557219E-02
c,1.7,1.329000E-03,-9.122000E-03,2.398700E-02,-5.448600E-02,3.332890E-01,5.401530E-01,
        2.575540E-01
c,6.6,1.0
c,7.7,1.0
d,P,3.026100E+00,6.204295E-01,2.107737E-01
f,P,4.771697E-01

s,S,1.271740E+01,7.211320E+00,4.108071E+00,2.297170E+00,1.100207E+00,4.758254E-01,
        2.126538E-01,9.413496E-02
c,1.8,1.735000E-03,-8.089000E-03,8.814300E-02,-3.418750E-01,-4.562200E-02,5.034010E-01,
        5.565790E-01,1.457610E-01
c,7.7,1.0
c,8.8,1.0
p,S,1.390646E+01,8.407031E+00,5.255629E+00,3.286285E+00,6.403901E-01,2.485725E-01,
        9.175393E-02
c,1.7,1.974000E-03,-1.109000E-02,2.356400E-02,-5.335700E-02,3.470450E-01,5.322760E-01,
        2.592810E-01
c,6.6,1.0
c,7.7,1.0
d,S,3.607259E+00,7.800180E-01,2.638650E-01
f,S,5.634499E-01
```

```
s,Cl,1.390166E+01,8.099804E+00,4.560530E+00,2.737296E+00,1.194114E+00,5.845004E-01,
        2.595187E-01,1.134861E-01
c,1.8,2.569000E-03,-7.665000E-03,1.012690E-01,-3.692210E-01,-4.401400E-02,5.185750E-01,
        5.588800E-01,1.388690E-01
c,7.7,1.0
c,8.8,1.0
p,Cl,1.638099E+01,1.024131E+01,6.404853E+00,4.006446E+00,7.985489E-01,3.084952E-01,
        1.121782E-01
c,1.7,2.486000E-03,-1.178300E-02,2.182600E-02,-5.337800E-02,3.565500E-01,5.285790E-01,
        2.568250E-01
c,6.6,1.0
c,7.7,1.0
d,Cl,4.382079E+00,9.824805E-01,3.297425E-01
f,Cl,7.101264E-01

s,Ar,1.489270E+01,8.737185E+00,4.922859E+00,3.075375E+00,1.497732E+00,6.819138E-01,
        3.112818E-01,1.381357E-01
c,1.8,-9.380000E-04,1.646700E-02,6.215900E-02,-3.759580E-01,-1.241600E-02,5.160160E-01,
        5.453520E-01,1.439860E-01
c,7.7,1.0
c,8.8,1.0
p,Ar,1.937247E+01,1.210980E+01,7.575727E+00,4.739667E+00,9.657799E-01,3.737167E-01,
        1.352415E-01
c,1.7,2.327000E-03,-9.304000E-03,1.331000E-02,-4.765200E-02,3.643430E-01,5.258920E-01,
        2.519590E-01
c,6.6,1.0
c,7.7,1.0
d,Ar,4.947375E+00,1.198435E+00,4.003559E-01
f,Ar,8.956061E-01
```

cc-pV(Q+d)Z-ccECP

```
s,Al,7.949914E+00,4.529029E+00,2.432129E+00,1.498216E+00,8.078694E-01,4.166727E-01,
        1.939421E-01,8.995064E-02,4.109861E-02
c,1.9,4.006000E-03,-1.946100E-02,8.612900E-02,-1.601450E-01,-1.811080E-01,5.199000E-02,
        4.505290E-01,5.295550E-01,1.531160E-01
c,7.7,1.0
c,8.8,1.0
c,9.9,1.0
p,Al,9.876031E+00,5.669519E+00,3.471873E+00,1.009537E+00,3.966336E-01,1.728821E-01,
        7.330786E-02,3.065228E-02
c,1.8,3.550000E-04,-2.650000E-03,2.661000E-03,-4.195600E-02,1.123410E-01,3.727120E-01,
        4.744470E-01,1.917390E-01
c,6.6,1.0
c,7.7,1.0
c,8.8,1.0
d,Al,1.808743E+00,4.437953E-01,1.953366E-01,7.965274E-02
f,Al,4.429014E-01,1.741183E-01
g,Al,3.991464E-01

s,Si,1.000301E+01,5.840344E+00,3.100974E+00,1.915116E+00,1.108635E+00,4.398912E-01,
        2.578032E-01,1.248379E-01,5.743548E-02
c,1.9,3.791000E-03,-1.650600E-02,8.843000E-02,-1.625180E-01,-1.976540E-01,8.836700E-02,
        4.405530E-01,5.132750E-01,1.497040E-01
c,7.7,1.0
c,8.8,1.0
c,9.9,1.0
p,Si,1.098641E+01,6.385523E+00,3.886146E+00,1.600543E+00,4.555836E-01,2.091416E-01,
        9.417660E-02,4.141521E-02
c,1.8,6.460000E-04,-4.084000E-03,6.136000E-03,-4.316700E-02,1.934680E-01,4.180540E-01,
```

```
        4.034270E-01,1.267590E-01
c,6.6,1.0
c,7.7,1.0
c,8.8,1.0
d,Si,2.496123E+00,6.300131E-01,2.729389E-01,1.126100E-01
f,Si,5.618072E-01,2.150865E-01
g,Si,4.981687E-01

s,P,1.144594E+01,6.702436E+00,3.643314E+00,2.230657E+00,1.278947E+00,4.990009E-01,
        2.941025E-01,1.542838E-01,7.338672E-02
c,1.9,3.636000E-03,-1.631400E-02,1.016420E-01,-2.236860E-01,-1.711240E-01,1.998080E-01,
        4.185340E-01,4.581190E-01,1.328930E-01
c,7.7,1.0
c,8.8,1.0
c,9.9,1.0
p,P,1.238219E+01,7.179106E+00,4.380560E+00,2.051358E+00,6.334161E-01,3.006345E-01,
        1.361396E-01,5.897863E-02
c,1.8,6.610000E-04,-4.157000E-03,5.708000E-03,-5.113500E-02,1.859600E-01,4.147800E-01,
        4.097020E-01,1.329160E-01
c,6.6,1.0
c,7.7,1.0
c,8.8,1.0
d,P,3.273202E+00,8.687763E-01,3.732263E-01,1.540900E-01
f,P,7.321927E-01,2.818867E-01
g,P,6.303479E-01

s,S,1.304422E+01,7.527028E+00,4.217795E+00,2.548020E+00,1.440352E+00,6.091360E-01,
        3.388140E-01,1.790031E-01,8.751556E-02
c,1.9,2.456000E-03,-1.244500E-02,1.062420E-01,-2.807790E-01,-1.395360E-01,2.534480E-01,
```

```
        4.626390E-01,4.018840E-01,1.061460E-01
c,7.7,1.0
c,8.8,1.0
c,9.9,1.0
p,S,1.371753E+01,8.019559E+00,4.884424E+00,2.754758E+00,7.559207E-01,3.478529E-01,
        1.550598E-01,6.663373E-02
c,1.8,1.109000E-03,-6.752000E-03,1.034900E-02,-5.156400E-02,2.331750E-01,4.287970E-01,
        3.736230E-01,1.132400E-01
c,6.6,1.0
c,7.7,1.0
c,8.8,1.0
d,S,3.776298E+00,1.071908E+00,4.629455E-01,1.902392E-01
f,S,8.914082E-01,3.265297E-01
g,S,7.092373E-01

s,Cl,1.407461E+01,8.237384E+00,4.734507E+00,2.858994E+00,1.509973E+00,6.958753E-01,
        3.820075E-01,2.194034E-01,1.095167E-01
c,1.9,2.929000E-03,-1.088200E-02,1.051570E-01,-3.325820E-01,-9.824000E-02,3.335220E-01,
        4.255470E-01,3.602330E-01,1.135250E-01
c,7.7,1.0
c,8.8,1.0
c,9.9,1.0
p,Cl,1.475695E+01,8.897799E+00,5.560378E+00,3.474530E+00,9.358483E-01,4.292111E-01,
        1.895415E-01,8.042236E-02
c,1.8,1.752000E-03,-1.041900E-02,1.703400E-02,-5.707600E-02,2.443960E-01,4.313190E-01,
        3.658380E-01,1.107200E-01
c,6.6,1.0
c,7.7,1.0
c,8.8,1.0
```

```
d,Cl,4.531908E+00,1.347215E+00,5.809417E-01,2.378593E-01
f,Cl,1.121156E+00,4.158898E-01
g,Cl,8.543884E-01

s,Ar,1.499224E+01,8.819630E+00,5.336511E+00,3.329262E+00,1.474522E+00,8.804411E-01,
        4.500511E-01,2.669738E-01,1.336571E-01
c,1.9,5.600000E-04,4.352000E-03,9.420400E-02,-3.560670E-01,-1.407840E-01,3.887130E-01,
        4.359850E-01,3.452360E-01,1.224840E-01
c,7.7,1.0
c,8.8,1.0
c,9.9,1.0
p,Ar,1.646851E+01,1.026891E+01,6.415053E+00,4.009512E+00,1.132865E+00,5.202681E-01,
        2.293103E-01,9.671475E-02
c,1.8,2.358000E-03,-1.051600E-02,9.491000E-03,-5.125700E-02,2.507430E-01,4.332380E-01,
        3.605570E-01,1.087320E-01
c,6.6,1.0
c,7.7,1.0
c,8.8,1.0
d,Ar,4.977088E+00,1.628626E+00,7.053144E-01,2.878835E-01
f,Ar,1.408786E+00,5.409325E-01
g,Ar,1.023726E+00
```

aug-cc-pV(D+d)Z-ccECP

```
s,Al,9.467632E+00,5.626780E+00,2.011750E+00,1.153557E+00,1.680911E-01,6.127412E-02,
        2.088086E-02
c,1.6,2.106000E-03,-5.525000E-03,6.608500E-02,-2.884150E-01,6.652750E-01,4.728490E-01
c,6.6,1.0
c,7.7,1.0
```

```
p,Al,5.086488E+00,3.178563E+00,1.986886E+00,1.961017E-01,5.681130E-02,1.477338E-02
c,1.5,-5.667000E-03,1.819200E-02,-2.883900E-02,4.918900E-01,6.154460E-01
c,5.5,1.0
c,6.6,1.0
d,Al,1.308217E+00,1.832422E-01,5.296992E-02

s,Si,1.108664E+01,6.688456E+00,2.621138E+00,1.512618E+00,2.341472E-01,8.553995E-02,
        3.051215E-02
c,1.6,2.178000E-03,-5.499000E-03,8.033500E-02,-3.226790E-01,6.862160E-01,4.647370E-01
c,6.6,1.0
c,7.7,1.0
p,Si,6.598755E+00,4.126387E+00,2.581579E+00,2.886592E-01,8.597700E-02,2.459099E-02
c,1.5,-5.591000E-03,2.022600E-02,-3.687200E-02,5.193070E-01,5.869270E-01
c,5.5,1.0
c,6.6,1.0
d,Si,1.738470E+00,2.675427E-01,8.004663E-02

s,P,1.238236E+01,7.485122E+00,3.082440E+00,1.926247E+00,3.056385E-01,1.119803E-01,
        3.879193E-02
c,1.6,2.195000E-03,-5.501000E-03,1.094400E-01,-3.638660E-01,6.998080E-01,4.593350E-01
c,6.6,1.0
c,7.7,1.0
p,P,8.332862E+00,5.213576E+00,3.265010E+00,3.917816E-01,1.186369E-01,3.398252E-02
c,1.5,-5.202000E-03,1.989200E-02,-4.200200E-02,5.369650E-01,5.687700E-01
c,5.5,1.0
c,6.6,1.0
d,P,2.161064E+00,3.604973E-01,1.116762E-01

s,S,1.371939E+01,8.361204E+00,3.780862E+00,2.363702E+00,3.851312E-01,1.408929E-01,
```

```
        4.709953E-02
c,1.6,2.159000E-03,-6.249000E-03,1.213550E-01,-3.861230E-01,7.155840E-01,4.512290E-01
c,6.6,1.0
c,7.7,1.0
p,S,1.075098E+01,6.729523E+00,4.218423E+00,5.045883E-01,1.473090E-01,4.041793E-02
c,1.5,-4.307000E-03,1.663900E-02,-3.974800E-02,5.441890E-01,5.668270E-01
c,5.5,1.0
c,6.6,1.0
d,S,2.624970E+00,4.600632E-01,1.449989E-01

s,Cl,1.468532E+01,9.081676E+00,4.517516E+00,2.823726E+00,4.726501E-01,1.727413E-01,
        5.666529E-02
c,1.6,3.796000E-03,-1.095200E-02,1.279250E-01,-3.953880E-01,7.245830E-01,4.471770E-01
c,6.6,1.0
c,7.7,1.0
p,Cl,1.328317E+01,8.318690E+00,5.220721E+00,6.312944E-01,1.821076E-01,4.898700E-02
c,1.5,-3.718000E-03,1.493300E-02,-3.997300E-02,5.513830E-01,5.617710E-01
c,5.5,1.0
c,6.6,1.0
d,Cl,3.195709E+00,5.780413E-01,1.901646E-01

s,Ar,1.561888E+01,9.399571E+00,5.294684E+00,3.311215E+00,5.648221E-01,2.065069E-01,
        6.623105E-02
c,1.6,1.705000E-03,-2.509000E-03,1.186860E-01,-4.024230E-01,7.324230E-01,4.447530E-01
c,6.6,1.0
c,7.7,1.0
p,Ar,1.648115E+01,1.031973E+01,6.477514E+00,7.679845E-01,2.212524E-01,5.755607E-02
c,1.5,-2.576000E-03,1.244000E-02,-3.908600E-02,5.569660E-01,5.563190E-01
c,5.5,1.0
```

```
c,6.6,1.0
d,Ar,3.640558E+00,7.022656E-01,2.353302E-01
```

aug-cc-pV(T+d)Z-ccECP

```
s,Al,8.212835E+00,4.555659E+00,2.241051E+00,1.298574E+00,6.706170E-01,2.208360E-01,
        9.829919E-02,4.356435E-02,1.670994E-02
c,1.8,2.969000E-03,-1.335600E-02,7.908900E-02,-2.317920E-01,-9.787600E-02,4.101350E-01,
        5.783300E-01,1.869940E-01
c,7.7,1.0
c,8.8,1.0
c,9.9,1.0
p,Al,8.744620E+00,5.193068E+00,2.982244E+00,1.387771E+00,2.586180E-01,9.758792E-02,
        3.679630E-02,1.155016E-02
c,1.7,9.110000E-04,-5.434000E-03,9.921000E-03,-3.063100E-02,2.871910E-01,5.444390E-01,
        3.013670E-01
c,6.6,1.0
c,7.7,1.0
c,8.8,1.0
d,Al,1.814977E+00,3.198831E-01,1.079784E-01,3.499576E-02
f,Al,2.899838E-01,9.958119E-02

s,Si,9.665766E+00,5.482739E+00,2.775639E+00,1.643690E+00,8.488039E-01,3.097429E-01,
        1.385535E-01,6.089588E-02,2.487397E-02
c,1.8,2.722000E-03,-1.138100E-02,9.174100E-02,-2.748170E-01,-9.523000E-02,4.248530E-01,
        5.844440E-01,1.850260E-01
c,7.7,1.0
c,8.8,1.0
c,9.9,1.0
```

```
p,Si,9.981886E+00,6.178782E+00,3.676271E+00,1.953541E+00,3.720789E-01,1.446960E-01,
        5.503535E-02,1.962433E-02
c,1.7,1.540000E-03,-7.908000E-03,1.617000E-02,-4.200400E-02,3.158970E-01,5.424010E-01,
        2.731420E-01
c,6.6,1.0
c,7.7,1.0
c,8.8,1.0
d,Si,2.391269E+00,4.580241E-01,1.552119E-01,5.475022E-02
f,Si,3.692667E-01,1.367250E-01

s,P,1.122502E+01,6.369909E+00,3.337544E+00,1.973366E+00,9.581721E-01,4.051031E-01,
        1.809439E-01,7.953374E-02,3.594531E-02
c,1.8,2.402000E-03,-9.789000E-03,9.596100E-02,-3.140620E-01,-8.159000E-02,4.443610E-01,
        5.855890E-01,1.805490E-01
c,7.7,1.0
c,8.8,1.0
p,P,1.181951E+01,6.805271E+00,4.141216E+00,2.587294E+00,4.999773E-01,1.978731E-01,
        7.557219E-02,2.629990E-02
c,1.7,1.329000E-03,-9.122000E-03,2.398700E-02,-5.448600E-02,3.332890E-01,5.401530E-01,
        2.575540E-01
c,6.6,1.0
c,7.7,1.0
c,8.8,1.0
d,P,3.026100E+00,6.204295E-01,2.107737E-01,7.798817E-02
f,P,4.771697E-01,1.716984E-01

s,S,1.271740E+01,7.211320E+00,4.108071E+00,2.297170E+00,1.100207E+00,4.758254E-01,
        2.126538E-01,9.413496E-02,3.685113E-02
c,1.8,1.735000E-03,-8.089000E-03,8.814300E-02,-3.418750E-01,-4.562200E-02,5.034010E-01,
```

```
        5.565790E-01,1.457610E-01
c,7.7,1.0
c,8.8,1.0
c,9.9,1.0
p,S,1.390646E+01,8.407031E+00,5.255629E+00,3.286285E+00,6.403901E-01,2.485725E-01,
        9.175393E-02,3.191429E-02
c,1.7,1.974000E-03,-1.109000E-02,2.356400E-02,-5.335700E-02,3.470450E-01,5.322760E-01,
        2.592810E-01
c,6.6,1.0
c,7.7,1.0
c,8.8,1.0
d,S,3.607259E+00,7.800180E-01,2.638650E-01,1.045166E-01
f,S,5.634499E-01,2.180021E-01

s,Cl,1.390166E+01,8.099804E+00,4.560530E+00,2.737296E+00,1.194114E+00,5.845004E-01,
        2.595187E-01,1.134861E-01,4.329746E-02
c,1.8,2.569000E-03,-7.665000E-03,1.012690E-01,-3.692210E-01,-4.401400E-02,5.185750E-01,
        5.588800E-01,1.388690E-01
c,7.7,1.0
c,8.8,1.0
c,9.9,1.0
p,Cl,1.638099E+01,1.024131E+01,6.404853E+00,4.006446E+00,7.985489E-01,3.084952E-01,
        1.121782E-01,3.891738E-02
c,1.7,2.486000E-03,-1.178300E-02,2.182600E-02,-5.337800E-02,3.565500E-01,5.285790E-01,
        2.568250E-01
c,6.6,1.0
c,7.7,1.0
c,8.8,1.0
d,Cl,4.382079E+00,9.824805E-01,3.297425E-01,1.371921E-01
```

```
f,Cl,7.101264E-01,3.135908E-01


s,Ar,1.489270E+01,8.737185E+00,4.922859E+00,3.075375E+00,1.497732E+00,6.819138E-01,
        3.112818E-01,1.381357E-01,4.594268E-02
c,1.8,-9.380000E-04,1.646700E-02,6.215900E-02,-3.759580E-01,-1.241600E-02,5.160160E-01,
        5.453520E-01,1.439860E-01
c,7.7,1.0
c,8.8,1.0
c,9.9,1.0
p,Ar,1.937247E+01,1.210980E+01,7.575727E+00,4.739667E+00,9.657799E-01,3.737167E-01,
        1.352415E-01,4.591860E-02
c,1.7,2.327000E-03,-9.304000E-03,1.331000E-02,-4.765200E-02,3.643430E-01,5.258920E-01,
        2.519590E-01
c,6.6,1.0
c,7.7,1.0
c,8.8,1.0
d,Ar,4.947375E+00,1.198435E+00,4.003559E-01,1.698676E-01
f,Ar,8.956061E-01,4.091794E-01
```

aug-cc-pV(Q+d)Z-ccECP

```
s,Al,7.949914E+00,4.529029E+00,2.432129E+00,1.498216E+00,8.078694E-01,4.166727E-01,
        1.939421E-01,8.995064E-02,4.109861E-02,1.578828E-02
c,1.9,4.006000E-03,-1.946100E-02,8.612900E-02,-1.601450E-01,-1.811080E-01,5.199000E-02,
        4.505290E-01,5.295550E-01,1.531160E-01
c,7.7,1.0
c,8.8,1.0
c,9.9,1.0
c,10.10,1.0
```

```
p,Al,9.876031E+00,5.669519E+00,3.471873E+00,1.009537E+00,3.966336E-01,1.728821E-01,
        7.330786E-02,3.065228E-02,1.017416E-02
c,1.8,3.550000E-04,-2.650000E-03,2.661000E-03,-4.195600E-02,1.123410E-01,3.727120E-01,
        4.744470E-01,1.917390E-01
c,6.6,1.0
c,7.7,1.0
c,8.8,1.0
c,9.9,1.0
d,Al,1.808743E+00,4.437953E-01,1.953366E-01,7.965274E-02,2.922625E-02
f,Al,4.429014E-01,1.741183E-01,6.441994E-02
g,Al,3.991464E-01,1.661437E-01

s,Si,1.000301E+01,5.840344E+00,3.100974E+00,1.915116E+00,1.108635E+00,4.398912E-01,
        2.578032E-01,1.248379E-01,5.743548E-02,2.365888E-02
c,1.9,3.791000E-03,-1.650600E-02,8.843000E-02,-1.625180E-01,-1.976540E-01,8.836700E-02,
        4.405530E-01,5.132750E-01,1.497040E-01
c,7.7,1.0
c,8.8,1.0
c,9.9,1.0
c,10.10,1.0
p,Si,1.098641E+01,6.385523E+00,3.886146E+00,1.600543E+00,4.555836E-01,2.091416E-01,
        9.417660E-02,4.141521E-02,1.635956E-02
c,1.8,6.460000E-04,-4.084000E-03,6.136000E-03,-4.316700E-02,1.934680E-01,4.180540E-01,
        4.034270E-01,1.267590E-01
c,6.6,1.0
c,7.7,1.0
c,8.8,1.0
c,9.9,1.0
d,Si,2.496123E+00,6.300131E-01,2.729389E-01,1.126100E-01,4.818720E-02
```

```
f,Si,5.618072E-01,2.150865E-01,8.743436E-02
g,Si,4.981687E-01,2.263078E-01

s,P,1.144594E+01,6.702436E+00,3.643314E+00,2.230657E+00,1.278947E+00,4.990009E-01,
        2.941025E-01,1.542838E-01,7.338672E-02,3.293226E-02
c,1.9,3.636000E-03,-1.631400E-02,1.016420E-01,-2.236860E-01,-1.711240E-01,1.998080E-01,
        4.185340E-01,4.581190E-01,1.328930E-01
c,7.7,1.0
c,8.8,1.0
c,9.9,1.0
c,10.10,1.0
p,P,1.238219E+01,7.179106E+00,4.380560E+00,2.051358E+00,6.334161E-01,3.006345E-01,
        1.361396E-01,5.897863E-02,2.262816E-02
c,1.8,6.610000E-04,-4.157000E-03,5.708000E-03,-5.113500E-02,1.859600E-01,4.147800E-01,
        4.097020E-01,1.329160E-01
c,6.6,1.0
c,7.7,1.0
c,8.8,1.0
c,9.9,1.0
d,P,3.273202E+00,8.687763E-01,3.732263E-01,1.540900E-01,6.591598E-02
f,P,7.321927E-01,2.818867E-01,1.080602E-01
g,P,6.303479E-01,2.562073E-01

s,S,1.304422E+01,7.527028E+00,4.217795E+00,2.548020E+00,1.440352E+00,6.091360E-01,
        3.388140E-01,1.790031E-01,8.751556E-02,3.551585E-02
c,1.9,2.456000E-03,-1.244500E-02,1.062420E-01,-2.807790E-01,-1.395360E-01,2.534480E-01,
        4.626390E-01,4.018840E-01,1.061460E-01
c,7.7,1.0
c,8.8,1.0
```

```
c,9.9,1.0
c,10.10,1.0
p,S,1.371753E+01,8.019559E+00,4.884424E+00,2.754758E+00,7.559207E-01,3.478529E-01,
        1.550598E-01,6.663373E-02,2.636426E-02
c,1.8,1.109000E-03,-6.752000E-03,1.034900E-02,-5.156400E-02,2.331750E-01,4.287970E-01,
        3.736230E-01,1.132400E-01
c,6.6,1.0
c,7.7,1.0
c,8.8,1.0
c,9.9,1.0
d,S,3.776298E+00,1.071908E+00,4.629455E-01,1.902392E-01,8.418609E-02
f,S,8.914082E-01,3.265297E-01,1.370203E-01
g,S,7.092373E-01,3.057057E-01

s,Cl,1.407461E+01,8.237384E+00,4.734507E+00,2.858994E+00,1.509973E+00,6.958753E-01,
        3.820075E-01,2.194034E-01,1.095167E-01,4.335384E-02
c,1.9,2.929000E-03,-1.088200E-02,1.051570E-01,-3.325820E-01,-9.824000E-02,3.335220E-01,
        4.255470E-01,3.602330E-01,1.135250E-01
c,7.7,1.0
c,8.8,1.0
c,9.9,1.0
c,10.10,1.0
p,Cl,1.475695E+01,8.897799E+00,5.560378E+00,3.474530E+00,9.358483E-01,4.292111E-01,
        1.895415E-01,8.042236E-02,3.189509E-02
c,1.8,1.752000E-03,-1.041900E-02,1.703400E-02,-5.707600E-02,2.443960E-01,4.313190E-01,
        3.658380E-01,1.107200E-01
c,6.6,1.0
c,7.7,1.0
c,8.8,1.0
```

```
c,9.9,1.0
d,Cl,4.531908E+00,1.347215E+00,5.809417E-01,2.378593E-01,1.063254E-01
f,Cl,1.121156E+00,4.158898E-01,2.159381E-01
g,Cl,8.543884E-01,3.787343E-01

s,Ar,1.499224E+01,8.819630E+00,5.336511E+00,3.329262E+00,1.474522E+00,8.804411E-01,
        4.500511E-01,2.669738E-01,1.336571E-01,5.071317E-02
c,1.9,5.600000E-04,4.352000E-03,9.420400E-02,-3.560670E-01,-1.407840E-01,3.887130E-01,
        4.359850E-01,3.452360E-01,1.224840E-01
c,7.7,1.0
c,8.8,1.0
c,9.9,1.0
c,10.10,1.0
p,Ar,1.646851E+01,1.026891E+01,6.415053E+00,4.009512E+00,1.132865E+00,5.202681E-01,
        2.293103E-01,9.671475E-02,3.742817E-02
c,1.8,2.358000E-03,-1.051600E-02,9.491000E-03,-5.125700E-02,2.507430E-01,4.332380E-01,
        3.605570E-01,1.087320E-01
c,6.6,1.0
c,7.7,1.0
c,8.8,1.0
c,9.9,1.0
d,Ar,4.977088E+00,1.628626E+00,7.053144E-01,2.878835E-01,1.284648E-01
f,Ar,1.408786E+00,5.409325E-01,2.948560E-01
g,Ar,1.023726E+00,4.517628E-01
```

References

(S1) Kaufman, V.; Martin, W. C. Wavelengths and Energy Level Classifications for the Spectra of Aluminum (Al_{I} through Al_{XIII}). *J. Phys. Chem. Ref. Data* **1991**, *20*, 775–

858.

(S2) Martin, W. C.; Zalubas, R. Energy Levels of Silicon, Si I through Si XIV. *J. Phys. Chem. Ref. Data* **1983**, *12*, 323–380.

(S3) Martin, W. C.; Zalubas, R.; Musgrove, A. Energy Levels of Phosphorus, P_I through P_{XV}. *J. Phys. Chem. Ref. Data* **1985**, *14*, 751–802.

(S4) Martin, W. C.; Zalubas, R.; Musgrove, A. Energy Levels of Sulfur, S_I Through S_{XVI}. *J. Phys. Chem. Ref. Data* **1990**, *19*, 821–880.

(S5) Radziemski, L. J.; Kaufman, V. Wavelengths, Energy Levels, and Analysis of Neutral Atomic Chlorine (Cl_I). *J. Opt. Soc. Am.* **1969**, *59*, 424.
